# Mathematical analysis of the relationship between glucose and sodium in a large Swedish cohort of critically ill patients

**DOI:** 10.1371/journal.pone.0360072

**Published:** 2026-10-21

**Authors:** Marcus Ewert Broman, Innas Forsal

**Affiliations:** Department of Clinical Sciences, Faculty of Medicine, Lund University, Sweden; Sri Ramachandra Institute of Higher Education and Research (Deemed to be University), INDIA

## Abstract

**Background:**

Hyperglycemia causes compensatory decrease of plasma sodium concentration by an influx of water from the cellular compartment. When the glucose level is corrected, the sodium concentration will follow and increase to its original level.

**Methods:**

A complex MATLAB script incorporating the Katz and Hillier correction formulas was developed to evaluate the impact of hyperglycemia on sodium levels and to analyze sodium shifts in 9,863 patients across 297,714 paired sodium-glucose measurements. Intensive care unit (ICU) mortality and morbidity data were extracted for the full cohort to assess outcomes.

**Results:**

95.3% of the glucose values (283,686) implied a correction factor for the measured sodium levels in the cohort. Correction of all sodium values for hyperglycemia did not change the adjusted ICU mortalities. Increased maximum sodium shift/24 h starting from 4.00–5.99 mmol/L was related to increased independent ICU mortality. Correction of sodium levels by using Katz and Hillier formulas for hyperglycemia did not change the adjusted ICU mortalities. 602/9,863 (6.1%) patients showed a sodium shift more than 8 mmol/L/24 h. 31 patients (5.1%) were missed without correction for hyperglycemia by the Katz formula and 54 patients (9.0%) were missed without correction by the Hillier formula.

**Conclusion:**

Failure to correct sodium for hyperglycemia misses 5–9% of patients with large sodium shifts (>8 mmol/L per 24 hours). Additionally, a sodium shift as low as 4.00–5.99 mmol/L per 24 hours is an independent risk factor for increased ICU mortality.

## Introduction

Elevated blood glucose is common in critically ill patients. Hyperglycemia causes a decrease in plasma sodium concentration through an influx of water from the intracellular to the extracellular compartment [1], leading to a reversible drop in measured sodium concentration [1,2]. When glucose levels are corrected, sodium concentration ideally rises back to its baseline level. However, in more slowly developing clinical states, such as diabetic ketoacidosis, extracellular volume is consistently depleted, meaning simple hemodilution does not entirely determine the final sodium concentration. Furthermore, a given glucose level can cause varying degrees of sodium lowering depending on whether blood glucose is trending upward or downward [3]. Because excessively rapid sodium shifts are harmful, managing hyperglycemia over time directly impacts decisions regarding external sodium administration [2].

The decrease in sodium concentration via water influx occurs as the body maintains osmotic equilibrium across cell membranes [4,5].

The Katz correction formula (Equation 1) estimates this inverse relationship between glucose and sodium. This theoretical formula is currently used in clinical practice to calculate reciprocal sodium shifts during hyperglycemia, applying to blood glucose values above a baseline of 5.6 mmol/L [1].


$$\begin{array}{c} orrectedNa=measuredNa+(\text{1}.\text{6}x(glucose-\text{5}.\text{6}))/\text{5}.\text{6} \end{array}$$ (1)

The Katz formula assumes an isolated increase in extracellular glucose while ignoring changes in total body water and other monovalent ions. It therefore models a simplified, closed system [1]. Our cohort consists of critical care patients who typically have no free oral intake and are subject to tightly controlled fluid balances. In contrast, patients with intact renal function and unconstrained fluid intake driven by thirst represent an open system, where osmotic diuresis and variable dilution introduce additional complexity [6–10].

In a follow-up study of six young, healthy subjects, Hillier et al. demonstrated that the slope of sodium decline deviates from linearity. According to the Hillier correction (Equation 2), a coefficient of 2.4 best models the sodium drop for glucose values exceeding 24 mmol/L (400 mg/dL) [10].


$$\begin{array}{c} orrectedNa=measuredNa+(\text{2}.\text{4}x(glucose-\text{5}.\text{6}))/\text{5}.\text{6} \end{array}$$ (2)

In clinical practice, the Katz and Hillier formulas account for auto-shifts in sodium levels during the treatment of hyperglycemia. Nevertheless, the measured sodium concentration at any given point must be treated as the true physiological level, as it represents the immediate osmolar environment surrounding the cells.

In critically ill patients, hyperglycemia often stems from pre-existing diabetes, acute stress responses, critical illness, corticosteroid administration, or parenteral nutrition/glucose infusions [9]. Co-occurring sodium disorders are also common in this population, making accurate sodium correction both critical and challenging [8–11]. Overcorrecting sodium risks severe brain tissue shrinkage or swelling, which can cause permanent neurological damage, particularly central pontine myelinolysis. Risk factors for myelinolysis include severe hyponatremia (Na < 105 mmol/L), prolonged hyponatremia, hypokalemia, chronic alcoholism, malnutrition, and advanced liver disease. Because active sodium administration and concomitant auto-correction via glucose lowering both contribute to the net rate of sodium change, current consensus guidelines recommend capping the total sodium shift at a maximum of 8 mmol/L per 24 hours [12].

This study aimed to: characterize hyperglycemia-induced sodium shifts in a large, real-world ICU cohort (n = 9,863 patients; 297,714 simultaneous sodium/glucose pairs extracted from electronic medical records, EMR) by using modern mathematical software, quantify how many patients experienced an unsafe sodium shift (>8 mmol/L per 24 h), evaluate the association between sodium shift magnitude and the adjusted ICU mortality, and assess whether applying the Katz versus Hillier formula corrections alters the observed impact on ICU mortality.

## Methods

All available patients (>18 years) treated at the general adult ICU at Skåne University Hospital, Lund, Sweden during 1/1/2006–31/12/2019 were included. The patients’ sodium results and glucose results were extracted from the ICCA electronic medical records (ICCA, Philips, Eindhoven, Netherlands) into excel files.

The inclusion criteria were: all patients during the time period of 1/1/2006–31/12/2019 over 18 years of age. There were no further exclusion criteria except younger age than 18 years.

The work for the large retrospective data extraction for the study started in 2017 and was finalized in 2020, and the large analysis was finalized in 2025. This study is retrospective.

A script in MATLAB 2022a (MathWorks^®^, Massachusetts, USA) was created with the following features; it could read all patient data and create a large raw material database containing all the patients’ clinical data which was anonymized, it could store the parameter label and the adjacent individual value and timestamp, it could carry out sorting, it could calculate any statistical parameter included in MATLAB, it could create hypotheses and compare them statistically, it could simulate all sodium values based on the Katz formula and on the Hillier formula, and it could draw graphs based on the data in the raw material database due to simulations and hypotheses.

Simultaneous Na-glucose pairs were created due to the timestamps of each Na-glucose result. A difference of maximum of 2 minutes was allowed.

In total 297,714 simultaneous Na-glucose pairs were obtained from 9,863 critically ill patients.

The time stamps for the deaths within the ICU were obtained, or status alive at the point of discharge from ICU, and this data was included in the raw material database in MATLAB.

Delta Na (ΔNa) was defined as the difference between two directly measured Na levels with different time stamps on the time line. A measured Na level is the prevailing Na level in the sample, which the analysis device reports. MATLAB did every possible subtraction of Na values over 24 hours and chose the largest difference.

The script was designed to scan for and detect the maximum ΔNa/24 hours in the Na profiles at any point of time during the ICU stay in the 5,022 patients in the cohort who stayed in ICU longer than 24 hours, and to analyze it.

All presented results and all figures were directly calculated and built from the clinical raw material in the cohort by the MATLAB script.

A multivariable Cox proportional hazards analysis model was also developed in MATLAB with the covariates age, sex, SOFA score day 1, SAPS3 score in order to evaluate the adjusted mortality of dysnatremias, of Katz and Hillier corrections and of maximum sodium shift over 24 hours at any point of time during the ICU stay.

Student’s t test, chi square (*X*^2^) test and Fisher’s exact test were all used for every significance calculation. All established glucose-sodium pairs were considered as independent values. The level for significance was set to p < 0.001. ICU mortalities are presented with CI 95%.

The sodium and glucose analyses were either carried out on a blood gas analyzer (Radiometer ABL 80, Radiometer, Copenhagen, Denmark) or at the Skåne University Hospital Clinical chemistry laboratory.

Permission for the study was obtained from the Swedish Ethical Review Authority (Dnr 2017/618). All methods were in accordance with relevant regulations and guidelines. Due to the retrospective nature of the study, the need to obtain the informed consent was waived by the Swedish Ethical Review Authority.

## Results

A total of 297,714 simultaneous sodium-glucose measurement pairs from 9,863 patients were analyzed. The linear relationships between measured and corrected sodium levels calculated via the Katz [1] and Hillier [10] formulas are displayed in Fig 1. Both formulas assume a baseline blood glucose level of 5.6 mmol/L (100 mg/dL), below which no sodium correction is applied.

**Fig 1:**
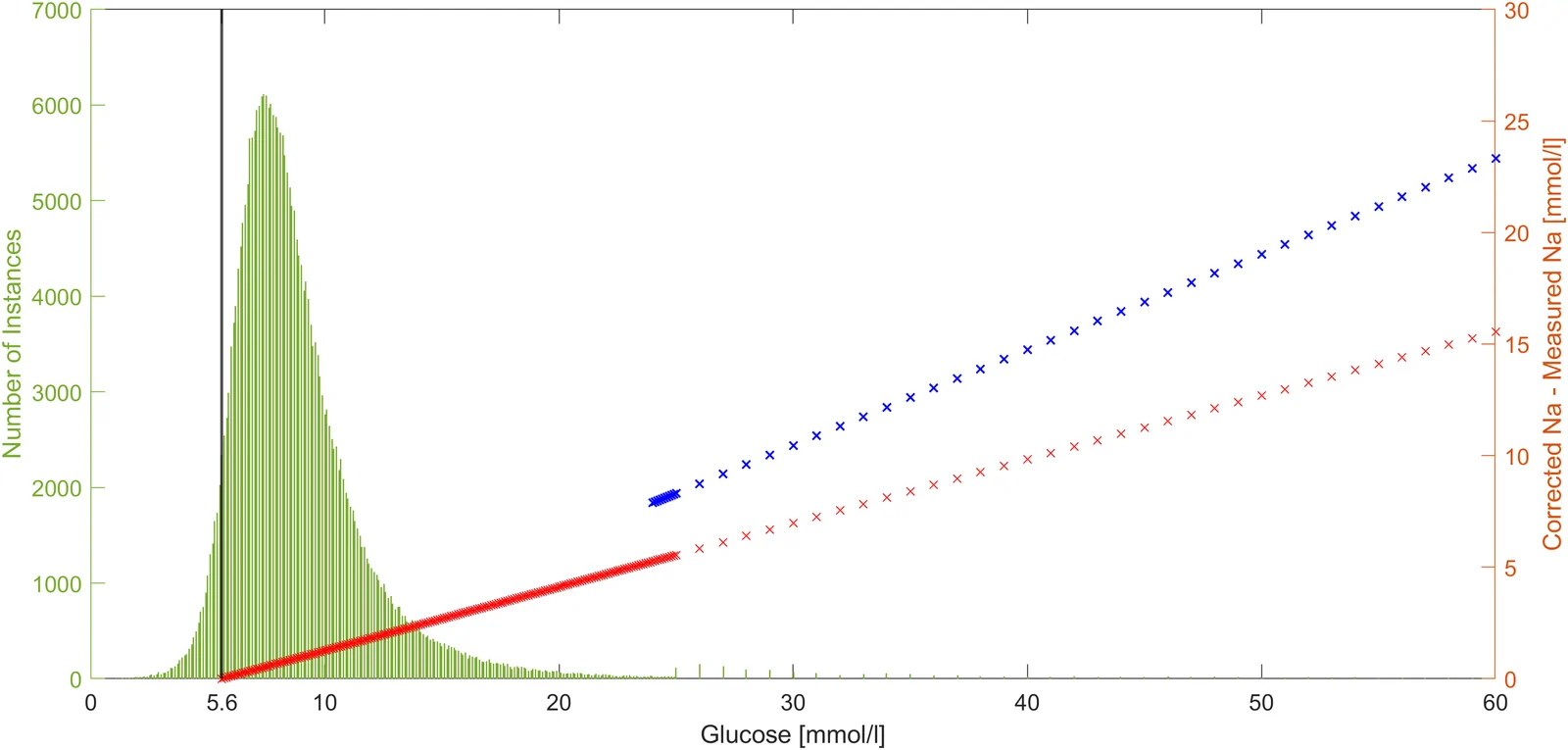
Corrections Katz Hillier. The relationship between glucose level and the difference: [corrected Na – measured Na]. The red linear graph shows the difference [corrected Na – measured Na] with increasing glucose level by using the correction formula of Katz (with a correction coefficient of 1.6, red graph). Hillier suggested a steeper correction of 2.4 for glucose values > 24 mmol/L, represented as a blue graph in the figure. Of note, glucose values >25 mmol/L were measured with an accuracy of ± 1 mmol/L and therefore this part of the lines is dotted. A histogram of all 297,714 real measured glucose values are inserted in the graph. For most (95.3%) of the glucose values a correction factor of the Na level is required.

Hyperglycemia requiring sodium correction (>5.6 mmol/L) was present in 95.3% (n = 283,686) of the paired samples. Only 4.7% (n = 14,028) of measurements in this critically ill cohort reflected normoglycemia or hypoglycemia <5.6 mmol/L (Fig 1).

Scatter plots and regression lines for uncorrected sodium versus values corrected by Katz (Fig 2) and Hillier (Fig 3) demonstrated significant divergence from uncorrected values at blood glucose levels >8.0 mmol/L (p = 0.02). While both formulas yield identical corrections between 5.6 and 23.9 mmol/L, their outputs diverge substantially at higher glucose concentrations (Fig 4). At a glucose level of 24.0 mmol/L, the discrepancy between formulas was 2.6 mmol/L (Katz correction: 5.3 mmol/L versus Hillier: 7.9 mmol/L). At 60.0 mmol/L, this discrepancy expanded to 7.8 mmol/L (Katz: 15.5 mmol/L versus Hillier: 23.3 mmol/L, respectively).

**Fig 2:**
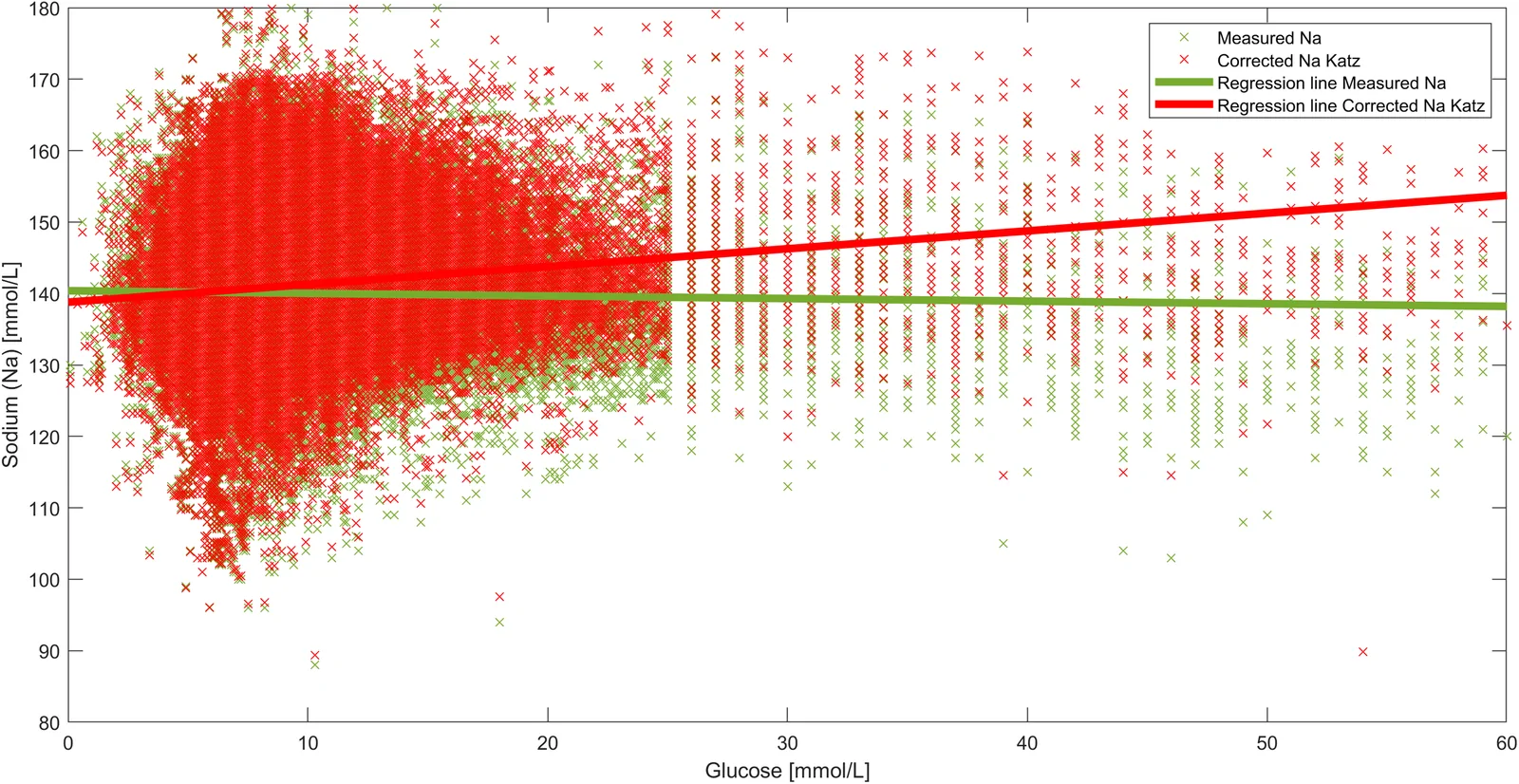
Katz vs measured. Plot of all measured sodium levels (colored green) and corresponding corrected sodium levels by Katz formula (colored red) with a correction factor of 1.6 for glucose levels above 5.6 mmol/L. Regression lines for the uncorrected sodium levels and the corrected levels by Katz were computed and compared. They differ significantly (p = 0.02) starting from glucose level 8 mmol/L (Students t test paired, Chi square and Fisher’s exact test).

**Fig 3:**
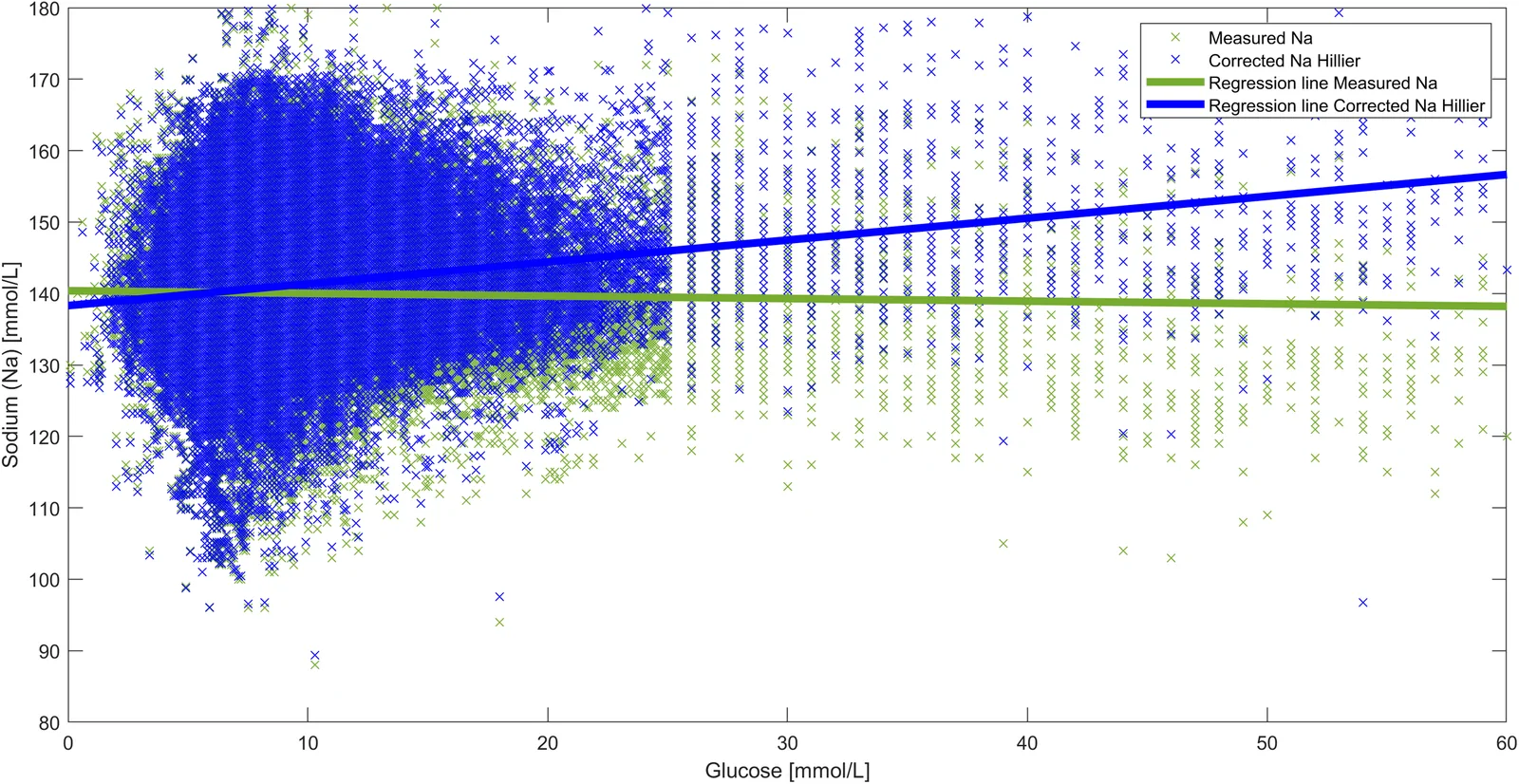
Hillier vs measured. Plot of all measured sodium levels (colored green) and corresponding corrected sodium levels by Hillier formula (colored blue) with a correction factor of 1.6 for glucose levels of 5.6–24 mmol/L, and a correction factor of 2.4 for higher glucose levels. Regression lines for the uncorrected sodium levels and the corrected levels by Hillier were computed and compared. They differ significantly (p = 0.02) starting from glucose level 8 mmol/L (Student’s t test paired, Chi square and Fisher’s exact test).

**Fig 4:**
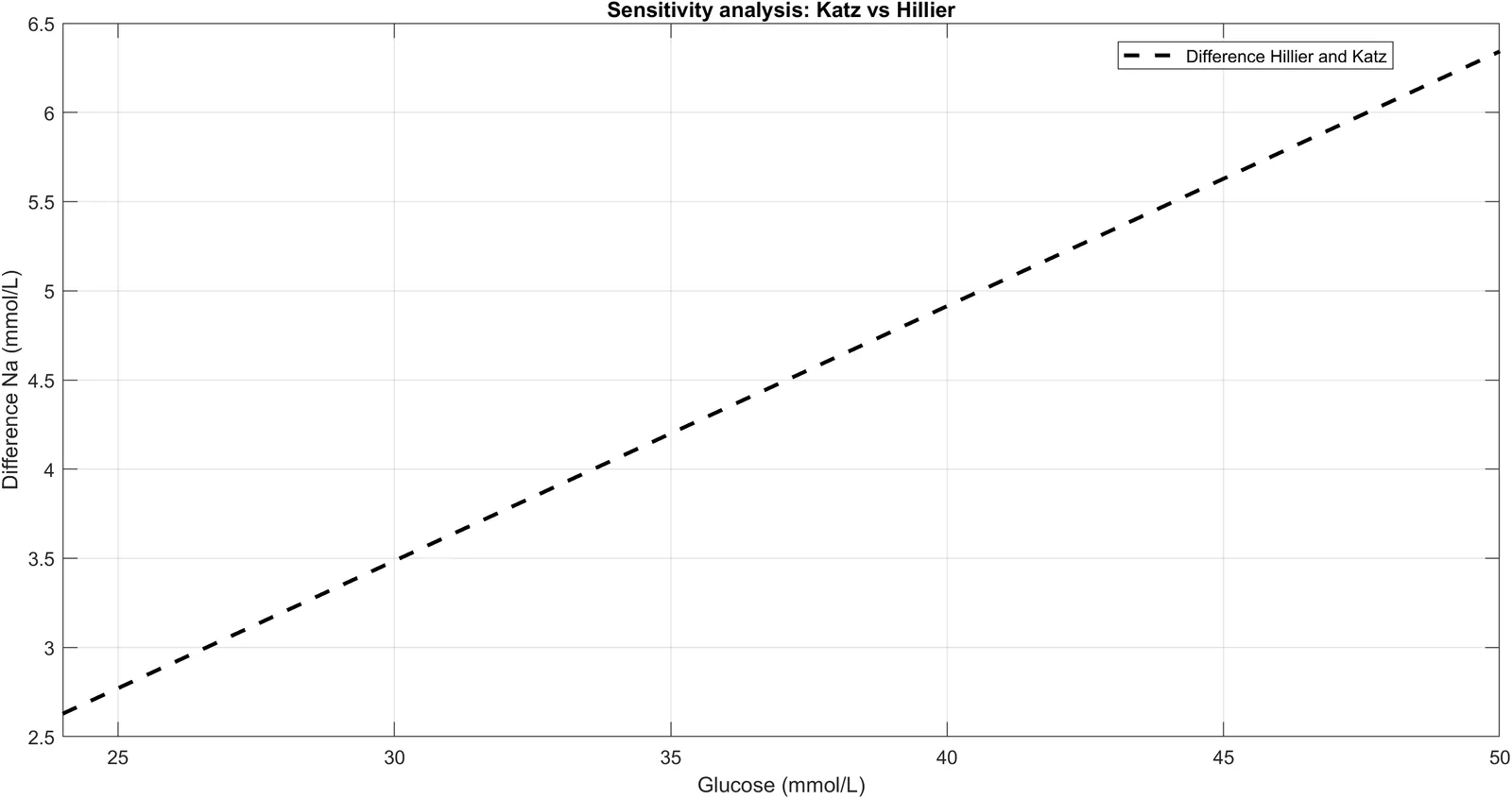
Sensitivity Katz versus Hillier difference. Sensitivity analysis between correction of sodium by using Katz simulation and Hillier simulation. Hillier suggests a stronger correction for glucose levels >24 mmol/L. The figure shows the difference between Katz and Hillier simulations.

In a multivariable Cox proportional hazards analysis adjusted for age, sex, day-1 SOFA score, and SAPS 3 score, progressive 24-hour sodium fluctuations ΔNa/24 h were significantly associated with increased ICU mortality, beginning at the 4.0–5.99 mmol/L category (Figs 5, 6 and Table 1). Absolute sodium ranges did not differ significantly across sodium shift categories (Table 1).

**Table 1:** An increasing ΔNa/24 h occurring at any time during the ICU stay was associated with higher ICU mortality. Across the overall cohort (n = 9,863; ICU mortality 13.1%), mortality differed significantly across all ΔNa/24 h categories (p < 0.001) and between individual categories (p < 0.001). In a multivariable Cox proportional hazards analysis adjusting for age, sex, day-1 SOFA score, SAPS 3 score, ΔNa/24 h remained a significant predictor of ICU mortality, driven by a significant increase in risk starting from category 4.00–5.99 mmol/L and up. Absolute sodium ranges did not differ significantly across sodium shift categories. Simulating sodium values using the Katz and the Hillier formulas for the adjacent glucose level did not significantly alter ICU mortality compared to uncorrected values. Significance testing was conducted by using Student’s t-test, Chi-square test, and Fisher’s exact test.

max ΔNa /24h	n uncorrected	ICU mortality uncorrected (95% CI)	hazard ratio (95% CI)	n Katz	ICU mortality Katz (95% CI)	hazard ratio (95% CI)	n Hillier	ICU mortality Hillier (95% CI)	hazard ratio (95% CI)	Na range uncorrected	Na range Katz	Na range Hillier
**0-** **1.99**	410	6.9 (4.4-9.4)		395	7.7 (5.1-10.3)		386	7.9 (5.2-10.6)		132.4 ± 6.9 to 147.5 ± 8.2	133.7 ± 6.7 to 148.6 ± 8.3	133.7 ± 6.7 to 148.8 ± 8.3
**2-** **3.99**	1105	9.4 (7.7-11.1)	1.18 (0.94-1.48) p = 0.143	1167	10 (8.3-11.7)	1.18 (0.94-1.49) p = 0.138	1135	9.9 (8.2-11.6)	1.07 (0.85-1.36) p = 0.524	132.5 ± 6.5 to 148.3 ± 8.2	133.7 ± 6.3 to 149.8 ± 8.3	133.8 ± 6.3 to 149.9 ± 8.3
**4-** **5.99**	1342	12 (10.3-13.7)	1.56 (1.18-2.06) p = 0.001	1397	13.6 (11.8-15.4)	1.77 (1.33-2.35) p < 0.001	1379	13.2 (11.4-15.0)	1.74 (1.32-2.30) p < 0.001	132.2 ± 6.2 to 150.0 ± 8.5	133.4 ± 6.0 to 151.6 ± 8.6	133.4 ± 6 to 151.7 ± 8.6
**6-** **7.99**	848	16.3 (13.8-18.8)	2.18 (1.49-3.19) p < 0.001	768	15.8 (13.2-18.4)	2.29 (1.50-3.51) p < 0.001	773	16 (13.4-18.6)	2.06 (1.33-3.17) p = 0.001	132 ± 6.2 to 151.7 ± 9.0	133.2 ± 6.4 to 153.4 ± 9.4	133.2 ± 6.4 to 153.4 ± 9.5
**8-** **9.99**	510	18.5 (15.1-21.9)	2.40 (1.22-4.71) p < 0.001	432	19.1 (15.4-22.8)	3.32 (1.65-6.30) p < 0.001	442	19.8 (16.1-23.5)	3.93 (2.23-6.92) p < 0.001	131 ± 7.5 to 152.6 ± 10.4	132.6 ± 7.9 to 154.6 ± 10.8	132.7 ± 7.9 to 154.7 ± 10.8
**10-** **12**	306	19.6 (15.2-24.1)	3.41 (1.39-8.34) p < 0.001	281	23.1 (18.2-28.0)	2.01 (0.52-8.39) p = 0.305	288	23.7 (18.8-28.6)	5.70 (2.51-12.93) p < 0.001	131 ± 8.8 to 154.8 ± 11.2	131.8 ± 9 to 154.9 ± 11.5	132 ± 9.0 to 155 ± 11.4
**>12**	105	31.7 (19.8-37.1)	10.2 (4.74-22.04) p < 0.001	153	36.4 (20.5-39.1)	10.41 (5.08-21.32) P < 0.001	169	33.1 21.0-34.5)	6.87 (3.02-15.61) p < 0.001	128 ± 12.7 to 156.4 ± 13.8	130.1 ± 13.0 to 158.9 ± 14.1	130.7 ± 13 to 159.5 ± 14.1

**Fig 5:**
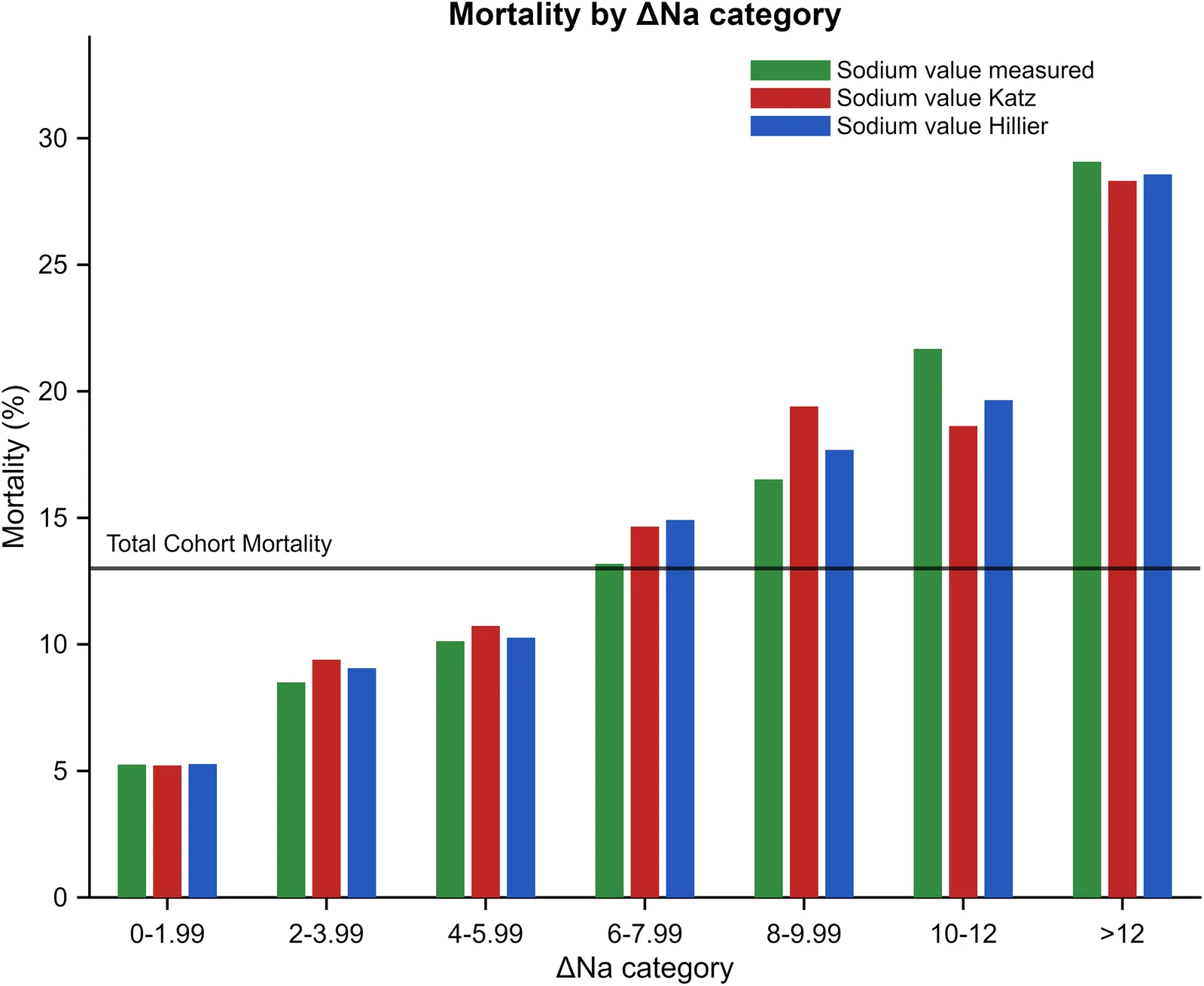
ICU mortality deltaNa. ICU mortality (%) across maximum ΔNa/24 h categories (largest sodium shift within 24 hours during ICU stay) for uncorrected sodium values (green), Katz-corrected values (red), and Hillier-corrected values (blue). Lower ΔNa/24 h categories were associated with lower mortality compared to the overall cohort mortality (13.1%, black reference line). ICU mortality increased with higher ΔNa/24 h shifts. No significant differences in mortality were observed between uncorrected sodium values and those corrected by the Katz or the Hillier formulas.

**Fig 6:**
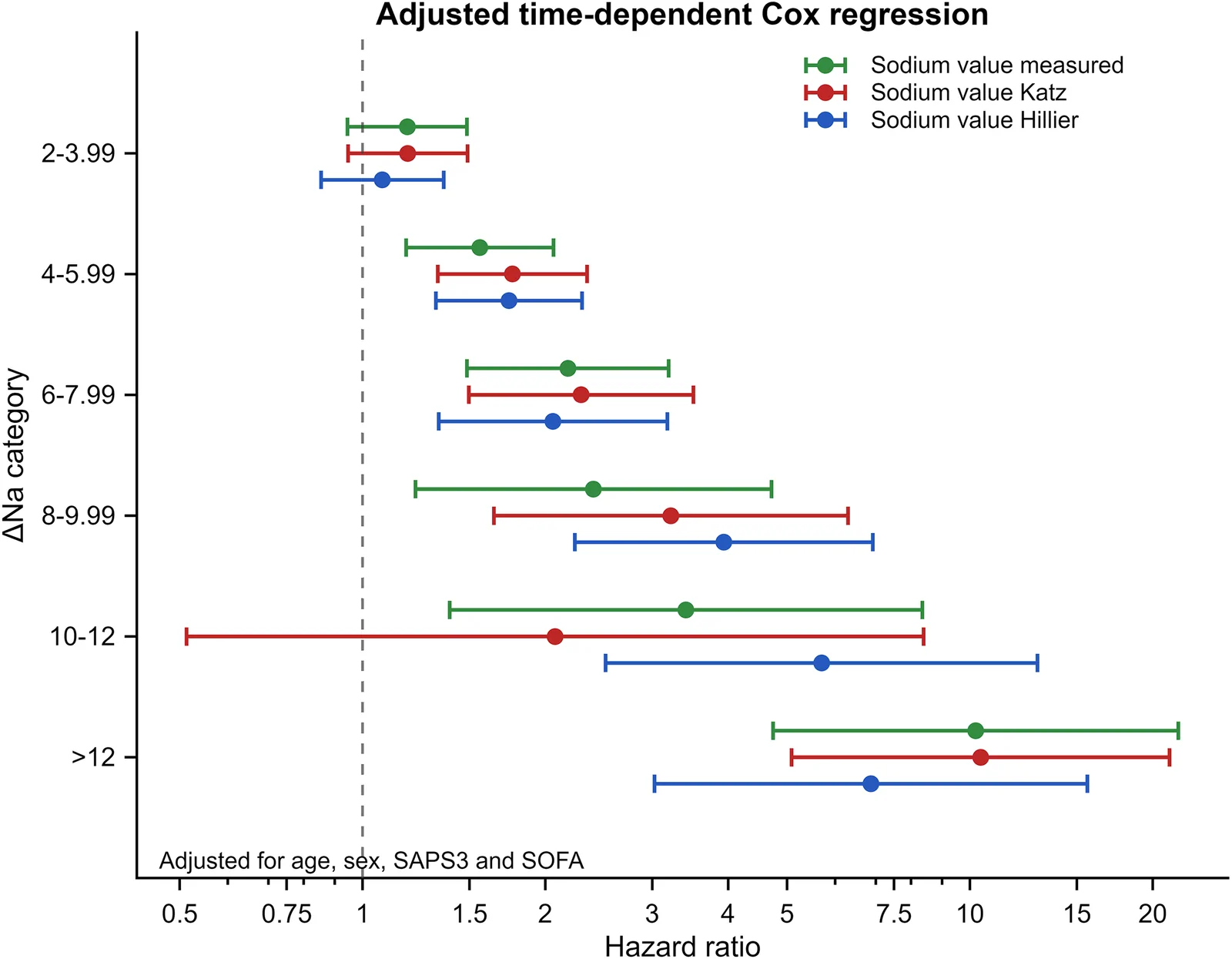
HR deltaNa. Hazard ratios for ICU mortality across ΔNa/24 h categories, adjusted for age, sex, day 1 SOFA score and SAPS3 score. ICU mortality increased significantly beginning at a sodium shift of 4–5.99 mmol/L/24 h. Hazard ratios did not differ significantly when using raw measured sodium values versus values transformed by the Katz or the Hillier formulas.

Multivariable evaluation of dysnatremias indicated that hypernatremia and an unstable sodium profile significantly increased ICU mortality, whereas hyponatremia did not (Table 2).

**Table 2:** ICU mortality across dysnatremia categories and sodium correction models. Dysnatremia categories were defined using uncorrected as well as corrected (Katz and Hillier formulas) serum sodium profiles: normonatremia; all Na values 135–145 mmol/L, hyponatremia (at least one Na < 135 mmol/L and none > 145 mmol/L, hypernatremia (at least one Na > 145 mmol/L and none <135 mmol/L), and unstable dysnatremia (at least one Na value <135 mmol/L and at least one > 145 mmol/L). Unadjusted hazard ratios demonstrated significantly higher ICU mortality in hypernatremia and unstable dysnatremia compared with normonatremia (p < 0.001), whereas hyponatremia showed no significant difference. Reclassifying sodium levels using Katz or Hillier glucose corrections did not significantly alter the associated hazard ratios or overall mortality risk distributions. Mortalities are presented as percentages with 95% confidence intervals. Group comparisons to normonatremia were evaluated using Student’s t-test for continuous variables and the Chi-square and Fisher’s exact test for categorical proportions. Unadjusted hazard ratios were derived from Cox proportional hazards regression model.

Na deviation	n	ICU mortality % (95% CI)	hazard ratio (95% CI)	n Katz	ICU mortality % Katz (95% CI)	hazard ratio (95% CI)	n Hillier	ICU mortality % Hillier	hazard ratio (95% CI)
**normonatremia** 135-145 mmol/L	4056	6.8 (6.0-7.6)		4110	6.5 (5.8-7.3)		4142	6.1 (5.4-6.9)	
**hyponatremia** at least one Na < 135 and no Na > 145	3913	11.3 (10.3-12.3)	1,41 (0.95-2.08) p = 0.087	3354	11.5 (10.4-12.6)	1.37 (0.92-2.04) p = 0.123	3011	11.6 (10.5-12.8)	1.68 (1.09-2.56) p = 0.191
**hypernatremia** at least one Na > 145 and no Na < 135	1186	17.7 (15.5-19.9)	2.50 (1.69-3.70) p < 0.001	1650	16.8 (15.0-18.6)	2.20 (1.50-3.22) p < 0.001	1943	16.5 (14.9-18.2)	2.46 (1.64-3.68) p < 0.001
**unstable** at least one Na < 135 AND at least one Na > 145	708	20.9 (17.9-23.9)	2.04 (1.34-3.12) p < 0.001	749	19.0 (16.2-21.8)	1.61 (1.05-2.49) p < 0.001	767	19.6 (16.8-22.4)	2.02 (1.28-3.18) p < 0.002

Correcting sodium values with either Katz or Hillier formula did not significantly alter overall ICU mortality or hazard ratios compared to uncorrected values (Tables 1, 2 and Figs 5, 6).

However, sodium correction significantly altered the identification of rapid sodium shifts. Based on uncorrected values, 602 patients exceeded the current consensus threshold of 8 mmol/L for ΔNa/24 h. Correction using the Katz formula identified 635 patients exceeding this threshold, indicating that uncorrected sodium missed 33 patients (5.1%; p = 0.02; Fig 7). Correction using the Hillier formula identified 656 patients, indicating that 54 patients (9.0%; p = 0.02; Fig 8) were missed without correction. The mean ±SD number of sodium measurements evaluated per 24-hour period was 7 ± 1.

**Fig 7:**
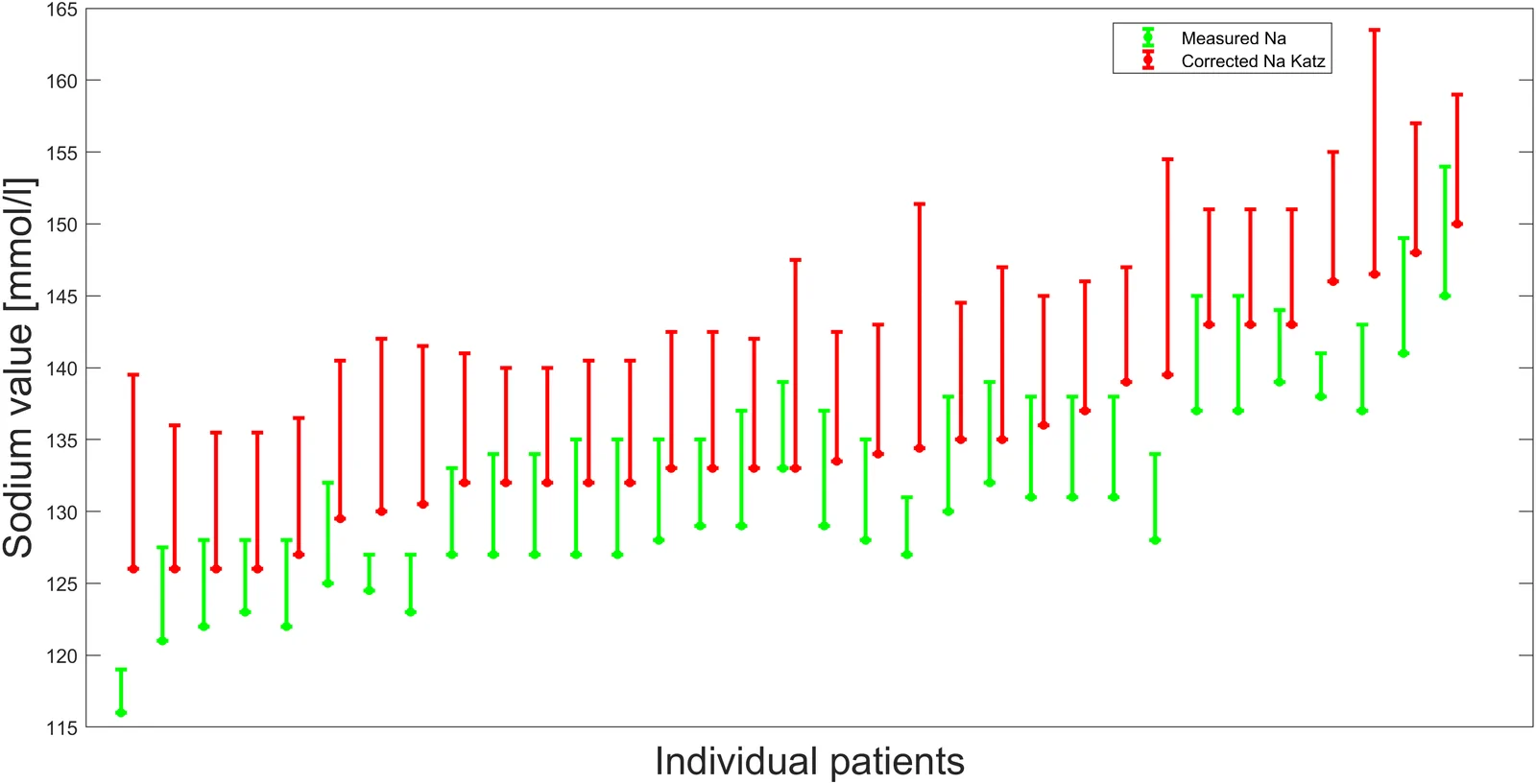
Measured Na versus Katz. Presentation of sodium changes (ΔNa/24 h) for the 33 patients in whom corrected Na levels by using the Katz formula (colored red) revealed a ΔNa > 8 mmol/L/24 h, which was not present when using the uncorrected Na levels (colored green).

**Fig 8:**
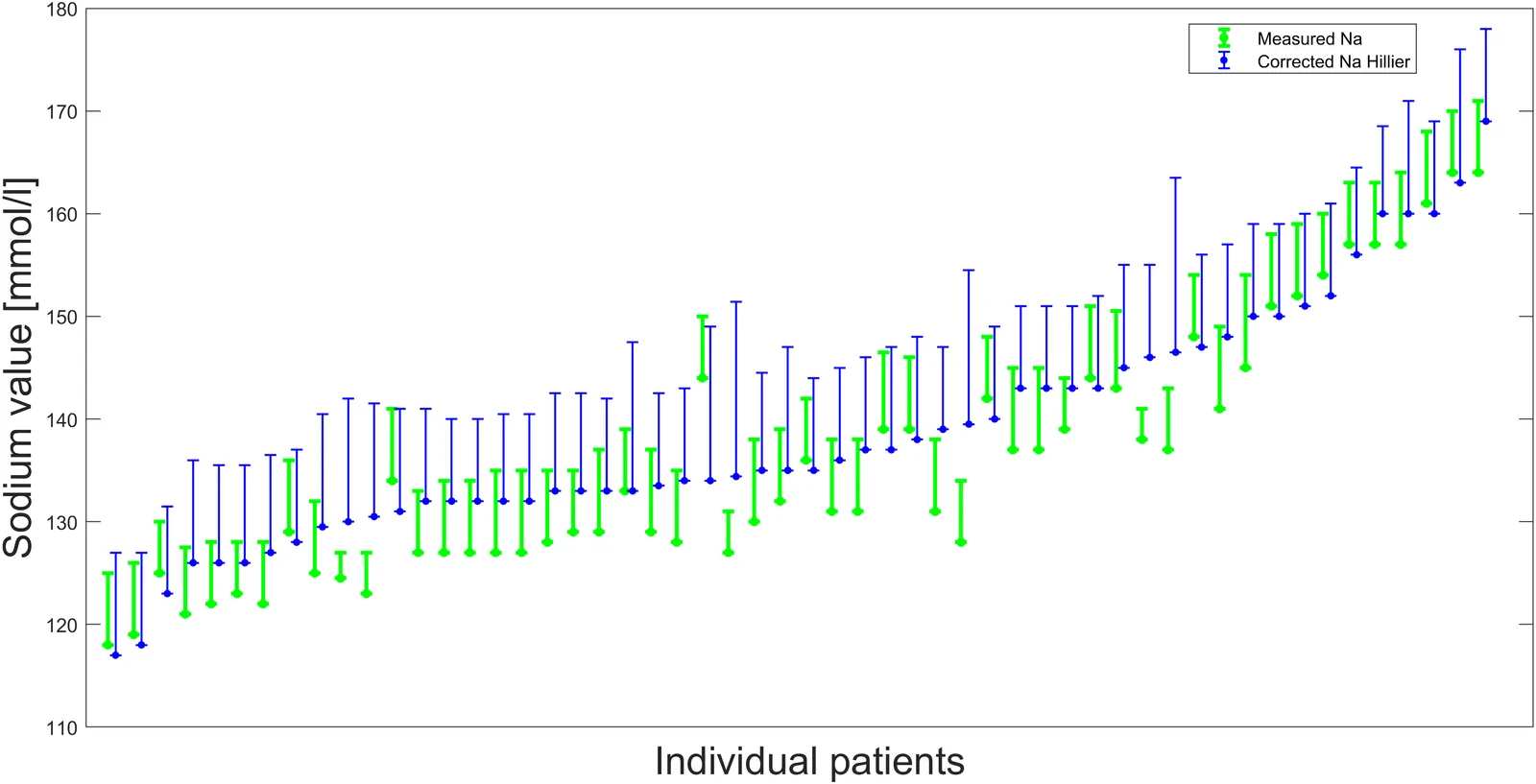
Measured Na versus Hillier. Presentation of ΔNa/24 h for the 54 patients in whom corrected Na levels by using the Hillier formula (colored blue) revealed a ΔNa > 8 mmol/L/24 h, which was not present when using the uncorrected Na levels (colored green).

## Discussion

In this investigation of 297,714 paired measurements in 9,863 ICU patients, we demonstrate two principal findings: first, uncorrected serum sodium fails to detect possible hazardous 24-hour sodium fluctuations (ΔNa/24 h > 8 mmol/L) in 5.1% to 9.0% of critically ill individuals, and second, daily sodium volatility as low as 4.00–5.99 mmol/L per 24 hours is an independent predictor of increased ICU mortality, establishing that sodium volatility as an independent risk factor.

Current consensus guidelines for managing dysnatremias recommend maintaining therapeutic sodium shifts at a maximum of 8 mmol/L per 24 hours to prevent osmotic demyelination syndrome and cerebral edema [12]. Our multivariable analysis, adjusted for age, sex, day 1 SOFA score, and SAPS 3 score, reveals that ICU mortality rises significantly at sodium shifts starting as low as 4.00–5.99 mmol/L per 24 hours (Table 1, Fig 6). Notably, this ICU mortality increase was driven by the rate of shift itself rather than the underlying absolute sodium concentration, as absolute sodium ranges remained comparable across sodium shift categories.

While absolute hypernatremia and fluctuating dysnatremia (mixed hyponatremia and hypernatremia) were independently tied to increased ICU mortality (Table 2), excess daily volatility also represents a distinct harm.

Hyperglycemia was present in 95.3% of all paired samples in this critically ill cohort, confirming that raw measured sodium rarely reflects baseline tonicity in the ICU. Measured sodium reflects the instantaneous extracellular tonicity surrounding cell membranes; however, as blood glucose is lowered during routine intensive care, water shifts back into the intracellular compartment, driving an auto-correction increase in measured sodium [1,10].

When comparing mathematical correction models, the classic Katz formula (1.6 mmol/L sodium drop per 5.6 mmol/L glucose elevation) and the non-linear Hillier model (2.4 factor above 24 mmol/L) both diverged significantly from uncorrected sodium values from blood glucose levels >8.0 mmol/L (p = 0.02). At extreme hyperglycemia (60.0 mmol/L), the divergence between them reached 7.8 mmol/L. While whole-cohort mortality hazards did not change when applying formula corrections, formula-based correction significantly improved single-patient safety tracking. Uncorrected sodium concealed unsafe shifts (>8 mmol/L per 24 h) in 33 patients (5.1%) under the Katz model and 54 patients (9.0%) under the Hillier model (Fig 7 and Fig 8).

Calculating corrected sodium values and tracking cumulative 24-hour shifts is cumbersome in busy intensive care environments. Given that failure to account for glucose shifts misses up to nearly 10% of patients exceeding safety thresholds, automated clinical decision support would be of help. Integrating auto-calculated corrected sodium values for instance on blood gas analysis results, and on electronic medical records (EMR) interfaces could assist the physician. Based on the non-linear osmotic dynamics described by Hillier [10] in severe hyperglycemia (>24 mmol/L), using the Hillier equation within automated EMR algorithms may provide superior safety margins against sodium (osmotic) overcorrection.

We consider the measured (prevailing) sodium to be the true physiological concentration and our target set point, as it reflects what cells actually experience. During hyperglycemia, measured sodium shifts automatically as glucose levels change (such as during treatment); larger glucose corrections induce larger sodium shifts. Although this automatic shift may carry lower risk for brain injury, overall sodium fluctuations must still be managed cautiously. The cumulative sodium change, combining auto-shifts and exogenous sodium administration, should not exceed 8 mmol/L in 24 hours [13–18].

The strengths of this study include its large sample size, high-density longitudinal data sampling (mean 7 measurements per 24 h), and robust multivariable adjustment for critical illness severity. Limitations include potential measurement noise because of retrospective EMR extractions from both blood gas analyzers and central laboratory systems [19]. Additionally, theoretical correction formulas model mostly closed physiological systems, whereas critically ill patients experience variable fluid balance, active resuscitation, and shifting renal function that introduce additional osmotic complexity. Different diagnoses (pathophysiologies) and the change in blood glucose over time might have an impact on the sodium level.

## Conclusion

A majority of the sodium values in this large ICU cohort implied a significant correction requirement for hyperglycemia. In our cohort 5–9% of the patients with dangerously high sodium shifts may be missed if correction of the sodium values by using Katz and/or Hillier formulas are not done. A sodium shift starting from 4–5.99 mmol/L causes an adjusted increase in ICU mortality, which implies the importance of monitoring sodium shifts over time in all patients.
